# Influence of Aqueous Solubility-Enhancing Excipients on the Microstructural Characteristics of Furosemide-Loaded Electrospun Nanofibers

**DOI:** 10.3390/pharmaceutics12040385

**Published:** 2020-04-23

**Authors:** Andrea Kovács, Adrienn Kazsoki, Balázs Démuth, Bernadett Szirányi, János Madarász, Károly Süvegh, Romána Zelkó

**Affiliations:** 1 Gedeon Richter Plc., Formulation R&D, Gyömrői Street 19-21, H-1103 Budapest, Hungary; kovacs.andrea@pharma.semmelweis-univ.hu (A.K.); demuth@oct.bme.hu (B.D.); b.sziranyi@richter.hu (B.S.); 2 University Pharmacy Department of Pharmacy Administration, Semmelweis University, Hőgyes Endre Street 7-9, H-1092 Budapest, Hungary; kazsoki.adrienn@pharma.semmelweis-univ.hu; 3 Department of Organic Chemistry and Technology, Budapest University of Technology and Economics, Budafoki út 8. 3, H-1103 Budapest, Hungary; 4 Department of Inorganic and Analytical Chemistry, Budapest University of Technology and Economics, Szent Gellért tér 4, H-1111 Budapest, Hungary; madarasz@mail.bme.hu; 5 Laboratory of Nuclear Chemistry, Eötvös Loránd University/HAS Chemical Research Center, P.O. Box 32, H-1518 Budapest, Hungary; suveghk@ceasar.elte.hu

**Keywords:** electrospinning, nanofiber, furosemide, morphology, physical-chemical characterization, microstructural distinction, positron annihilation lifetime spectroscopy

## Abstract

Electrospun nanofibers were prepared from furosemide-containing hydroxypropyl cellulose and poly(vinylpyrrolidone) aqueous solutions using different solubility enhancers. In one case, a solubilizer, triethanolamine, was applied, while in the other case a pH-modifier, sodium hydroxide, was applied. Scanning electron microscopy (SEM) was carried out for morphological characterization of the fibers. The SEM images indicated similar mean diameter size of the two fibrous formulations. However, in contrast to the NaOH-containing fibers of normal diameter distribution, the triethanolamine-containing fibers showed approximately normal diameter distribution, possibly due to their plasticizing effect and the consequent slightly ribbon-like morphology. Attenuated total reflectance-Fourier transform infrared spectroscopy (ATR-FTIR), powder X-ray diffraction (XRD) and positron annihilation lifetime spectroscopy (PALS) were applied for microstructural characterization. The FTIR measurements confirmed that furosemide salt was formed in both cases. There was no sign of any crystallinity based on the XRD measurements. However, the PALS highlighted the differences in the average o-Ps lifetime values and distributions of the furosemide-loaded fibrous formulations. The two types of electrospun nanofibrous formulations containing amorphous furosemide salt showed similar macrostructures but different microstructural characteristics depending on the type of solubility enhancers, which lead to altered storage stability.

## 1. Introduction

Furosemide is a loop diuretic which is used to treat different edematous diseases. As a drug from the fourth class of the biopharmaceutical classification system (BCS IV), it has low intestinal permeability and low water solubility at the physiological pH range [1]. In addition to the narrow absorption window and the intestinal efflux proteins further decrease its bioavailability [2]. Khandavilli et al. prepared high-solubility crystalline hydrates of Na and K furosemide salts to improve the poor solubility behavior [3], while other researchers have created several alternative drug carriers, like supramolecular complexes [4,5], hot-melt extrusion products [6], nanocapsules [7], microspheres [8] and halloysites [9] to overcome the unfavorable drug features. Drug-loaded nanofibrous delivery systems could be a promising alternative in case of furosemide, offering unique properties which contribute to the increase of bioavailability with a highly porous structure and a high surface-area-to-volume ratio, and enabling the preservation of the initially crystalline drug in amorphous form in the fibers, either in solid dispersion or in solid solution [10,11,12]. The most frequently used technique is electrospinning to obtain nanofibers; however, many different methods can be used. The characteristic diameter size of fibers is in the nano- and micrometer range [13,14,15].

Multifluid processes can greatly expand the capability of electrospinning in generating new types of nanostructures with different organizations of the inner parts, and from both spinnable and unspinnable working fluids [16]. However, multifluid electrospinning has more experimental parameters for influencing the formation of complex nanostructures compared with traditional single-fluid blending electrospinning. Among them, some new parameters will make the size prediction of nanofibers from a multifluid process complicated, for example, the compatibility between different fluids, their flow rates and relative ratios, their interfacial tensions, and their electrospinnability [17]. In order to finely tune the complex structure, effective tracking of the functionality-related structural elements is essential.

Besides the process parameters, the applied fiber-forming excipients determine the function of the final fibrous drug delivery system (DDS). Solubilizers are often used in pharmaceutical formulations to improve the aqueous solubility of drugs with poor solubility. On the other hand, solubilizers can influence the mechanical properties of the system by acting as plasticizers, which can modify the characteristics of the drug delivery system advantageously or disadvantageously [18,19]. They can provide an improved dissolution rate for the drugs, enhance wettability and help to avoid the precipitation of the active compound, but they also can contribute to the better physical stability of the formulation by reducing the recrystallization of the drug [12,20]. Panda et al. studied the effects of different plasticizers, including triethanolamine, on the crystallization of the drug in polymeric films. They found that, among the studied plasticizers, triethanolamine most increased the amorphization rate of the drug in the examined formulations [21]. However, sometimes, the use of surfactants causes the opposite effect, and it leads to recrystallization of the drug during storage in the amorphous solid dispersion (ASD) caused by the supersaturation of the formulation fibers [22], or enhanced molecular mobility, which is crucial in ASDs [23]. The effect of nanoscale confinement on the drug release properties of polymer nanofibers was investigated by analysis of the molecular mobility and drug release kinetics of cellulose acetate/sulindac nanofibers. The sulindac molecules were well dispersed in the amorphous polymer matrix while retaining their chemical integrity. ^1^H solid-state NMR analysis proved that the amorphous chains formed a highly packed structure when the diameter of the drug-loaded nanofibers decreased. In addition, sulindac release from the nanofibers was significantly retarded with increasing activation energy for both the main chain and the side chain motion of nanofibers, suggesting that the confinement-induced chain packing could lead to a slowing of drug release [24]. This means that an appropriate or inappropriate choice of excipients can be advantageous or disadvantageous regarding the stability and processability of fibers [22].

A comprehensive microstructural study with an examination of the supramolecular interactions of the compounds of the formulations [18] provides essential information about the prediction of the functional properties of the system. Special concern is required in the case of furosemide for the crystalline or amorphous state of the drug, the identification of amorphous acid or the amorphous salt state, which have particular relevance for stability prediction for the systems during storage [25].

The primary purpose of the present study was to track the supramolecular changes of the fibrous systems caused by the excipients applied for solubility enhancement of the formulations. Therefore, macro- and microstructural comparison were carried out to distinguish even slight differences in the furosemide-loaded fibrous systems, which may alter their functionality-related characteristics including their long-term stability.

## 2. Materials and Methods

### 2.1. Materials

Furosemide (FUR, 330.74 g/mol, >98% purity, Sigma-Aldrich, St. Louis, USA) was used as a model drug from the BCS IV class possessing acidic chemical groups, and thus changing its solubility as a function of actual pH value.

Hydroxypropyl cellulose (HPC, Klucel EXF Pharm, Mw~80,000, Ashland, Covington, KY, USA) and poly(vinylpyrrolidone) (PVP, Kollidon 90 F, Mw ~1,000,000–1,500,000, BASF, Ludwigshafen, Germany) were chosen as polymers. For the dissolution of the drug, triethanolamine (TEA, Ph. Eur.8.0., Molar Chemicals, Budapest, Hungary) was used as surfactant or sodium hydroxide (NaOH, Ph.Eur.8.0., Molar Chemicals, Budapest, Hungary) aqueous solution of 2 M concentration was used as a pH modulator, and purified water was used as solvents of the polymers.

### 2.2. Preparation of Furosemide-Containing Solutions for Electrospinning

Two different solutions were made for the electrospinning process. The composition of the first viscous solution was based on the authors’ previous study [11]. The stock solution, containing 1 *w/w*% furosemide, 1.5 *w*/*w*% triethanolamine used as a solubilizer, and purified water as a solvent, was used to prepare a homogeneous solution with magnetic stirring in a dark beaker. The dissolution of the drug was achieved within a few minutes; the pH value of the resulting solution was 8.4 ± 0.2 in every sample preparing. The preparation of a viscous polymeric solution was followed by adding 15 *w*/*w*% of the polymers in a ratio of 3/2 HPC/PVP at room temperature (25 °C). Firstly, the PVP was added to the solution under stirring, and, after the complete dissolution of the PVP, the HPC was added in small portions, and it was stirred for hours to reach a homogenous viscous solution (**composition-TEA**).

The second polymeric solution was defined based on our preliminary experiments. The stock solution also contained 1 *w*/*w*% of the drug and purified water was used as a solvent, by adding NaOH solution during magnetic stirring until total dissolution of the furosemide had been reached; the pH value of the resulting solution was 9.8 ± 0.2 in each prepared sample. Based on our previous study [11], the HPC/PVP *w*/*w* ratio was determined at 3/2; on the other hand, enhancement of the total polymer concentration was necessary for proper electrospinning. Thus, the total polymer concentration was chosen as 17 *w*/*w*% (**composition-NaOH**). The addition of the polymers was similar in each preparation.

The compositions of the two polymeric viscous solutions are summarized in Table 1.

### 2.3. Preparation of Polymeric Solutions for the Unloaded Electrospun Fibers

As references for solid-state characterization of drug-loaded fibers, neat fibers were prepared from similar polymeric solutions without active pharmaceutical ingredient (API). However, some changes were necessary to make solutions with the required properties for electrospinning. The amounts of the applied NaOH and triethanolamine were changed to keep the pH of the solutions at the same value as in the case of the corresponding furosemide-containing solutions. A decreased amount of NaOH and triethanolamine was applied, with dilution until the same pH values were achieved. After that, the polymers were added to the aqueous solutions. The total polymer concentration was also slightly changed. In the case of the solubilizer-containing solution, PVP was added to obtain a solution of 17 *w*/*w*% of total polymer concentration for successful electrospinning. In the case of neat composition-TEA, triethanolamine was added to reach pH 8.4 ± 0.2, followed by 15 *w*/*w*% of the polymers in a ratio of 3/2 HPC/PVP at room temperature (25 °C). Firstly, the PVP was added to the solution under stirring, and after the complete dissolution of the PVP, the HPC was added in small portions to the solution. It was stirred for hours to produce a homogenous viscous solution, and, finally, another 2 *w*/*w*% PVP was added to the solution. Meanwhile, in the case of neat composition-NaOH, an aqueous NaOH solution was diluted to reach pH 9.8 ± 0.2, and the further steps of the preparation were the same as in the corresponding drug-loaded composition-NaOH.

### 2.4. Preparation of Physical Mixtures

For Fourier-transform infrared spectroscopy (FTIR), X-ray diffraction (XRD) and positron annihilation lifetime spectroscopy (PALS) measurements, the powder physical mixtures of the viscous solution of composition-TEA were prepared and homogenized by stirring for about 5 min in a porcelain mortar; in the case of composition-NaOH, the physical mixtures were prepared similarly, but without NaOH. For the PALS measurements, it was necessary to press tablets with direct pressing of 0.2 g of the prepared powder by 1 ton for 20 s.

### 2.5. Formulation of Electrospun Nanofibers (NF)

The nanofibers were prepared by electrospinning with the electrospinning instrument (eSpinCube, SpinSplit LLC., Budapest, Hungary). The previously prepared viscous solutions were filled into syringes and connected with a teflon tube to the metallic needle (22G, inner diameter 0.70 mm). The prepared nanofibers were collected on a grounded aluminum collector. For sample preparation for the SEM and FTIR measurements, the collector was covered with aluminum foil, while, in case of the PALS and XRD measurements, parchment paper was used instead of aluminum foil, for easier sample removal. For optimal electrospinning of the first solubilizer-containing solution (composition-TEA) the electrospinning parameters were the following: 14 cm collector–needle distance, 18 kV applied voltage, 0.15 μL/s flow rate (the prepared furosemide-containing nanofibers: NF-FUR-TEA). In the case of the second polymeric solution without solubilizer (composition-NaOH): 13 cm collector–needle distance, 19 kV applied voltage, 0.15 μL/s flow rate (the prepared furosemide-containing nanofibers: NF-FUR-NaOH). The applied parameters were changed in the case of the preparation of neat fibers to the following: applied voltage: 12 kV, flow rate: 0.1 μL/s; the collector–needle distances were 14.5 cm in the case of composition-TEA (the prepared unloaded nanofibers: NF-PLACEBO-TEA) and 14 cm in the case of composition-NaOH, respectively. The sample preparation took place at 22 ± 1 °C and 43 ± 3% relative humidity (the prepared unloaded nanofibers: NF-PLACEBO-TEA).

### 2.6. Characterization of Electrospun Nanofibers

#### 2.6.1. Morphological Study by Scanning Electron Microscopy (SEM)

The morphology of the unstored nanofibers was examined by JEOL 6380LVa (JEOL, Tokyo, Japan) scanning electron microscope, using the secondary electron imaging detection method. The collected nanofibrous samples on aluminum foil were fixed with conductive double-sided carbon adhesive tape, and their surface was covered by sputtered gold before the SEM measurements. During the measurements, 15 kV accelerating voltage was applied and the working distance was 13 mm. The stored samples were examined by JEOL 5610LV (JEOL, Tokyo, Japan) scanning electron microscope using secondary electron imaging detection method and the measurements were carried out by similar sample preparation, with 15 kV accelerating voltage and 15 mm working distance.

#### 2.6.2. Statistical Analysis of the Fiber Diameters

The SEM images were used for the measurement, and the fiber diameters were calculated using the Image J program (US National Institutes of Health); 150 measurements from the fibrous elements were carried out in each SEM image, and the corresponding fiber diameters were determined. The diameter-size distribution of the samples was determined using the SPSS 20.0 software package (SPSS Inc., Chicago, IL, USA). The distribution of the fiber diameters was tested using the Kolgomorov−Smirnov test, and analysis of variance was done to determine the differences between the samples.

#### 2.6.3. Microstructural Evaluation of the Fibers by ATR-FTIR (Attenuated Total Reflectance—Fourier Transform Infrared) Spectroscopy

The ATR-FTIR spectra of the prepared fibers, the physical mixtures and the substances were measured using a Jasco FT/IR-4200 spectrophotometer (Jasco Inc., Easton, MD, USA), which was equipped with Jasco ATR PRO470-H single reflection accessory. The spectra were collected over a wavenumber range of 4000 and 400 cm^−1^. The measurements were performed in absorbance mode. After 100 scans, the measurements were evaluated with Spectra Manager-II, Jasco software. The absorbance data were normalized between 0 and 1 for better comparison of the different spectra.

#### 2.6.4. X-Ray Diffraction (XRD)

X-ray diffraction patterns were registered by X’pert Pro MDP X-ray diffractometer (PANalytical, Amelo, The Netherlands). Cu-K *α*-radiation (1.54 Å) and Ni filters were used; the applied voltage was 40kV, and the current was 30 mA. The samples were examined between 2°–42° 2*θ*. Intensity data were normalized between 0 and 1, before graphical display in OriginPro 8 software (OriginLab Corp., Northampton, MA, USA).

#### 2.6.5. Positron Annihilation Lifetime Spectroscopy (PALS)

A carrier-free ^22^NaCl positron source was used during the PALS measurements, with approximately 10^6^ Bq activity; the source was closed between Kapton foils. The samples were placed onto the two sides of the positron source and wrapped into aluminum foil. Three parallel measurements were carried out in each case. The spectra were collected with a fast-fast coincidence system with BaF_2_/XP2020Q detectors and Ortec electronics. The discrete lifetime values were evaluated using the RESOLUTION computer code [26], while a version of the MELT code [27] was used for distribution data analysis.

During the analysis, three lifetime components were observed; from these, the second and the third were chosen to further analysis. While the second component is related to the positron lifetime, the third, which is the longest lifetime, is related to annihilation of *ortho*-positronium atoms.

#### 2.6.6. Accelerated Stability Test

For the accelerated stability test, the prepared nanofibrous samples (furosemide-containing and placebo nanofibers in both formulations) were collected on parchment paper, then packed into aluminum foil and put into a plastic bag with hermetic closure. The samples were stored under controlled conditions (40 ± 2 °C, 75 ± 5% relative humidity) in a stability chamber (Sanyo type 022, Leicestershire, UK) for 4 weeks. After 1, 2, 3 and 4 weeks of storage the samples were further examined by SEM, FTIR and XRD.

#### 2.6.7. Furosemide Content of the Nanofibers

FUR content of the fibers was measured by Jasco 530 UV−vis Spectrophotometer (Agilent 8453 UV−vis Diode Array System, Agilent Technologies, Santa Clara, CA, USA) at 277 nm. FUR standard stock solution of 0.5 mg/mL was prepared in a calibrated volumetric flask with 0.1 M NaOH solution (Ph.Eur.8). Solutions of 5 concentrations were used for the calibration. The solutions with different concentrations of FUR were prepared by diluting the stock solution with pH 6.8 phosphate buffer (0.025 mol/dm^3^ KH_2_PO_4_), in a 0.0005−0.0075 mg/mL concentration range.

#### 2.6.8. In-Vitro Drug Release Study

The dissolution method was based on a previous study of our research group [18]; a small-volume dissolution method was used in order to mimic in-vivo buccal dissolution.

The fibrous samples were put into a sinker along with a stirring bar, and then it was placed into 20 mL of tempered dissolution medium. The temperature was set at 37 ± 1 °C using a water bath, and the stirring rate was 50 rpm. The dissolution was followed for 10 min, and 200 μL of samples were taken from the stirred medium at predetermined time intervals and diluted to 2.5 mL with the buffer for spectrophotometric analysis. Three parallel measurements were carried out, and the amount of the dissolved drug was compared to the theoretical drug content of the nanofibrous samples.

## 3. Results and Discussion

### 3.1. Morphology Analysis

**Figure 1:**
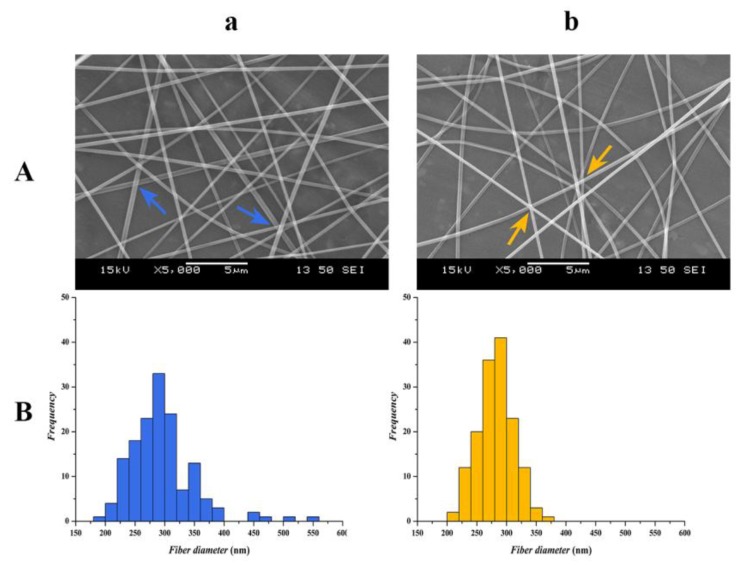
Scanning electron microscopic (SEM) images (**A**) and fiber diameter distributions (**B**) of the two compositions: (**a**) composition-TEA; (**b**) composition-NaOH.

Figure 1A illustrates the SEM images of nanofibers prepared from the two types of polymeric solutions. The results indicate that homogeneous fibers with some beads were obtained from both formulations.

The composition-TEA and composition-NaOH formulations have similar average fiber diameters (295 ± 54 nm and 281 ± 31 nm respectively), but the composition-NaOH shows a more homogenous structure with a smaller standard deviation.

The statistical analysis revealed differences between the two formulations. The diameter distribution of the composition-TEA formulation, containing TEA, was approximately normal (*p* = 0.048), and, in the case of composition-NaOH fibers (formulated from sodium hydroxide-containing solution), the normality was clearly confirmed (*p* = 0.909) by Kolmogorov–Smirnov test. One-way variance analysis showed that the two samples significantly differed from each other (*p* = 0.041). Figure 1B illustrates the distributions of the fiber diameters for the two formulations.

**Figure 2:**
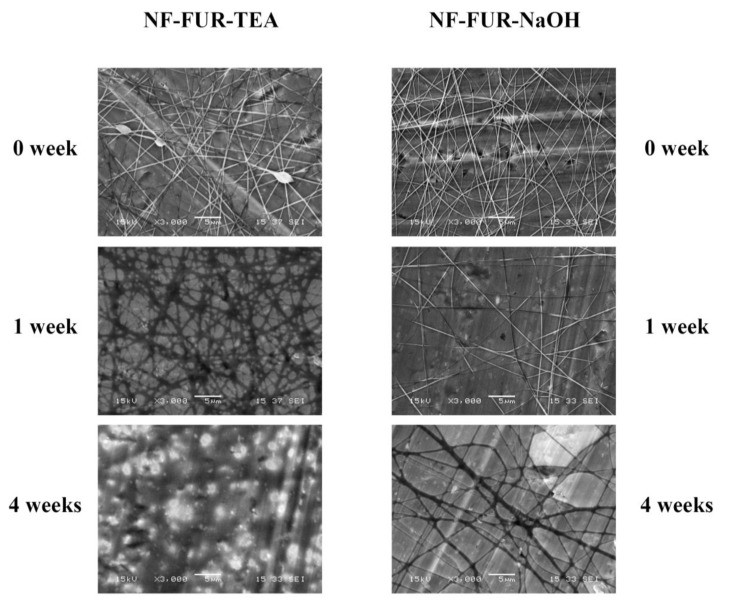
Scanning electron microscopic (SEM) images of the fibrous samples without storage and after 1 and 4 weeks of storage in the cases of NF-FUR-TEA and NF-FUR-NaOH.

A slight difference can be visually observed between the two compositions. Composition-TEA (Figure 1A/a) showed flat ribbon-like fiber segments at the contact points of individual fibers, which indicate the fiber-to-film transition initiated by the storage (Figure 2). During storage, this transition is proceeding, indicating the plasticizing feature of the applied solubilizer [21]. Meanwhile, the unstored sample of composition-NaOH (Figure 1A/b) formed from sodium hydroxide solution represented uniform fibers of cylindrical shape. The stored samples (Figure 2) of composition-NaOH also went through fiber widening and merging, but these changes were less dominant compared to the composition-TEA.

After the first week, the composition-NaOH remained in nearly the same state as at the beginning of the storage, while the composition-TEA had already changed.

After 4 weeks of storage, the fibrous structure of composition-TEA completely disappeared, and a continuous film was formed. The composition-NaOH preserved its fibrous structure with widened, flattened and partly merged fibers.

### 3.2. Solid-State Characterisation

#### 3.2.1. X-Ray Diffraction Patterns of the Fibers

**Figure 3:**
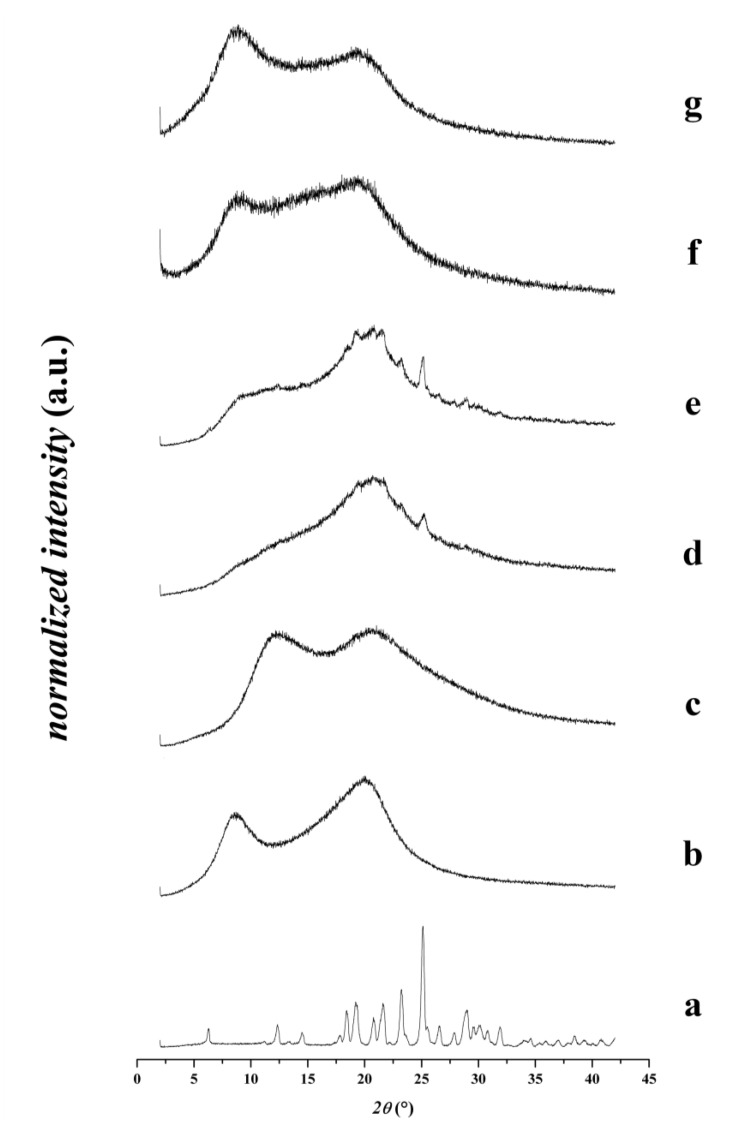
X-ray diffractograms of various samples: **a**—furosemide, **b**—HPC powder substance, **c**—PVP powder substance, **d**—physical mixtures of composition-TEA, **e**—physical mixtures of composition-NaOH without NaOH, **f**—nanofibrous formulation of composition-TEA, **g**—nanofibrous formulation of composition-NaOH.

The X-ray diffraction phase analysis showed that the pure crystalline furosemide has several sharp characteristic peaks. These confirm that the furosemide crystallized as Form I in triclinic structure [28,29]. The X-ray diffractograms of the physical mixtures clearly indicate the presence of crystalline furosemide, while the characteristic sharp peaks are missing in both nanofibrous formulations (Figure 3), which suggests the amorphous state of the active pharmaceutical ingredient.

#### 3.2.2. FTIR Spectroscopic Analysis

**Figure 4:**
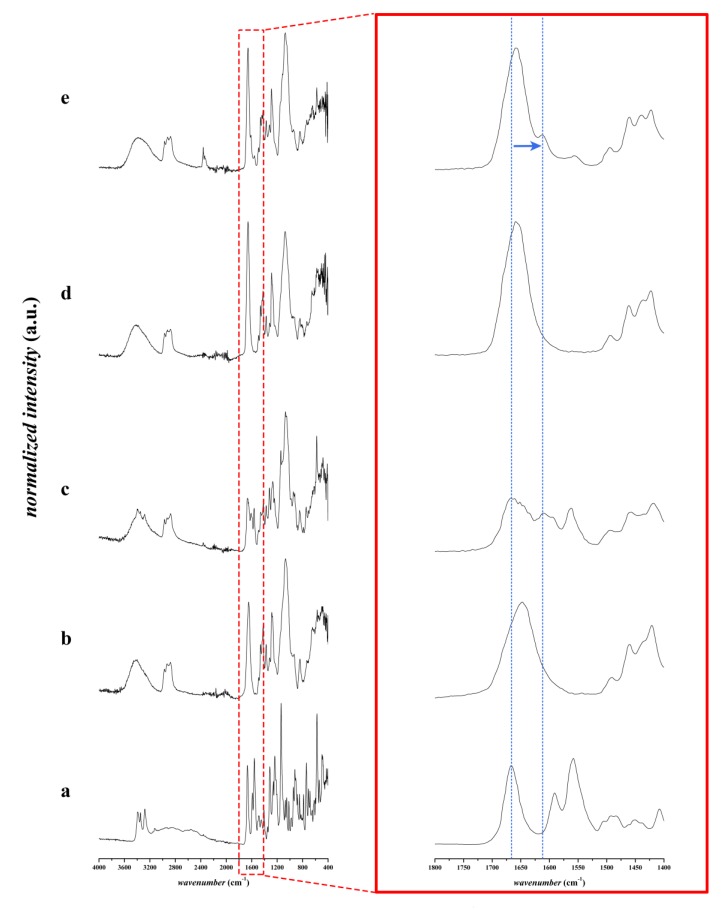
FTIR spectra of triethanolamine-containing compositions from 400 to 4000 cm^−1^ of **a**: furosemide, **b**: physical mixture of composition-TEA without furosemide, **c**: furosemide-containing physical mixture of composition-TEA, **d**: neat nanofibers of composition-TEA **e**: furosemide-containing nanofibers of composition-TEA; and the corresponding FTIR spectra in 1400–1800 cm^−1^ range. The shifting peak in the fingerprint region is shown with the blue dashed line and the arrow.

**Figure 5:**
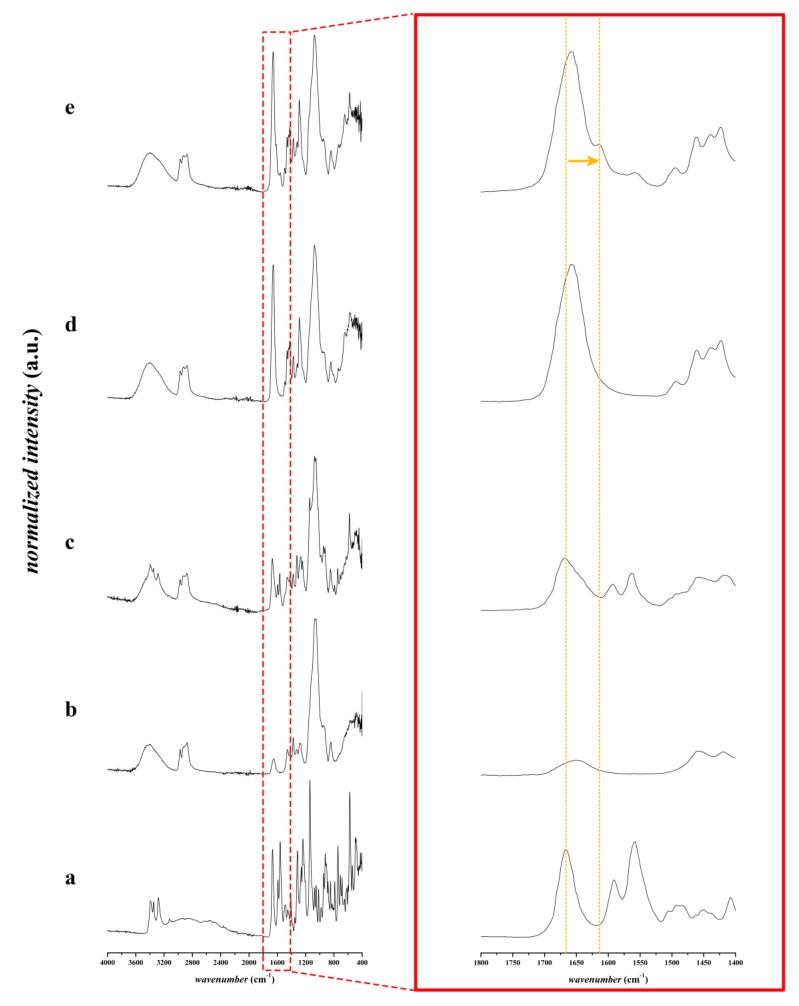
FTIR spectra of NaOH-containing composition from 400 to 4000 cm^−1^ of **a**: furosemide, **b**: physical mixture of composition-NaOH without furosemide and without NaOH, **c**: furosemide-containing physical mixture of composition-NaOH without NaOH, **d**: neat nanofibers of composition-NaOH **e**: furosemide-containing nanofibers of composition-NaOH; and the corresponding FTIR spectra in 1400–1800 cm^−1^ range. The shifting peak in the fingerprint region is shown with the orange dashed line and the arrow.

The FTIR spectra of the furosemide, the different physical mixtures and the corresponding fibrous structure are displayed in Figure 4 and Figure 5. The furosemide-characteristic peaks of NH stretching vibration of the sulphonamide (3395 cm^−1^ in pure furosemide, 3397 cm^−1^ in physical mixtures), NH stretching vibration of the secondary amine group (3348 cm^−1^ in pure furosemide, 3349 cm^−1^ in physical mixtures), and NH stretching vibration of sulphonamide (3281 cm^−1^ in pure furosemide, 3285 cm^−1^ in physical mixtures) [29,30] have disappeared in both nanofibrous samples containing furosemide. The FTIR spectra results are in a good agreement with the XRD results regarding the crystallinity of the samples. Based on the FTIR spectra, there is no evidence of the presence of furosemide in neutral acidic form in the nanofibrous formulations, in contrast to the physical mixture of the applied polymers and the furosemide.

On the other hand, we can observe some shifting peaks in the fingerprint region of the spectra. Nielsen et al. found a similar phenomenon in the case of spray drying of furosemide using an aqueous solution of sodium hydroxide in comparison with methanol as a solvent [25]. They reported that, in the first case, amorphous salt was formed because of the applied sodium hydroxide, while, from the methanolic solution of furosemide, an amorphous free acid was formed. They observed the C=O peak at 1670 cm^−1^ in the case of the amorphous free acid of the furosemide, while the characteristic peak at 1608 cm^−1^ refers to the formation of its amorphous salt. In our examinations, the C=O peak was at 1659 cm^−1^ in the case of composition-TEA, while it was shifted to 1657 cm^−1^ in composition-NaOH. In contrast to the physical mixture of the polymers and furosemide, this C=O peak appears at 1667 cm^−1^. The peaks at 1657 cm^−1^ and 1659 cm^−1^ also appeared in the corresponding neat fibers, so these signals refer to the C=O group of the PVP polymer. These characteristic peaks appeared at 1613 cm^−1^ in the case of composition-TEA and 1614 cm^−1^ in the case of composition-NaOH, respectively. The latter refers to the amorphous salt form of furosemide. However, it can also be observed at 1610 cm^−1^ in the physical mixture of composition-TEA, where the liquid triethanolamine was added to the powder excipients, and the mixing of the compositions in a mortar led to partial salt formation. Similar results were found by Abraham et al. They observed a new band at 1612 cm^−1^, corresponding to the COO^−^ group, while furosemide and triethanolamine were used for salt formation [31].

#### 3.2.3. Positron Annihilation Lifetime Spectroscopy

**Figure 6:**
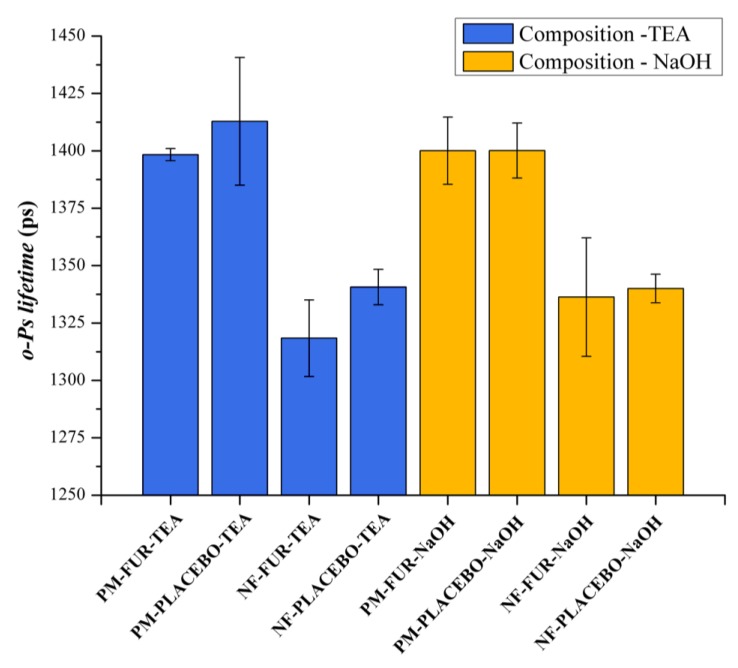
Average *o*-Ps lifetime values (±SD) of the physical mixture and nanofibers of two different compositions.

**Figure 7:**
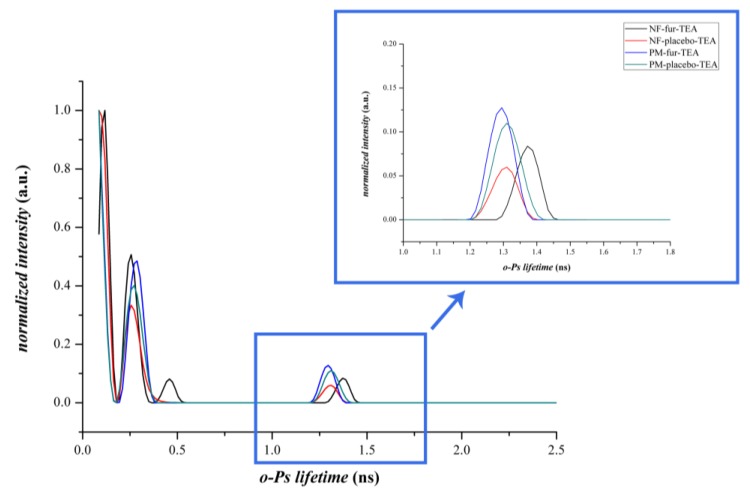
Distribution of *o*-Ps lifetimes of the physical mixture and nanofibers of composition-TEA and the corresponding placebo.

**Figure 8:**
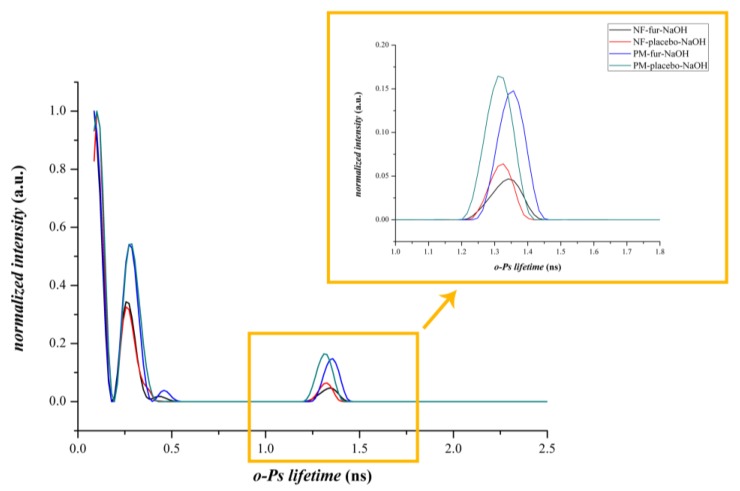
Distribution of *o*-Ps lifetimes of the physical mixture and nanofibers of composition-NaOH and the corresponding placebo.

PALS measurements were used to track the microstructural changes of the different compositions. The data from the PALS measurements visualized in Figure 6 did not show differences between the physical mixtures of composition-TEA and composition-NaOH, since the discrete *o*-Ps lifetime values were within the limit of standard deviations and the corresponding fibrous formulations were also similar. However, in the case of composition-TEA, the *o*-Ps values were slightly higher than in the case of composition-NaOH. On the other hand, the fibrous formulations and the corresponding physical mixtures were over standard deviation limits. The decrease of the *o*-Ps lifetimes indicated the formation of smaller average free volume holes, thus a denser supramolecular structure of the nanofibrous samples caused by different polymer–drug interactions [32]. A remarkable phenomenon was observed in connection with lifetime distribution data (Figure 7 and Figure 8). Intensity values belonging to the second, shorter-lifetime components (positron) and those of the third, longer-lifetime (*o*-Ps) values were also compared to obtain information about any possible structural changes and interactions between the applied excipients. In the case of the nanofibrous formulation of composition-TEA, the difference between the second and third lifetime components was much higher than in the case of composition-NaOH. This phenomenon may relate to the sulphonamide group of the furosemide, which is acting like a positronium inhibitor. In the formulation of composition-TEA, the positronium inhibitor effect is more pronounced, possibly because of the presence of the triethanolamine, which can contribute to a micellar structure formation of furosemide in the nanofibers [33].

Table 2 summarizes the characteristic values of the *o*-Ps lifetime distributions of various samples. The higher the *o*-Ps lifetime, the lower the local electron density of the system; thus, the obtained different average *o*-Ps values between the drug-loaded and the neat fibers reflect the differing supramolecular arrangements of the polymeric fibers. A difference was found in the *o*-Ps lifetime values of the two types of furosemide-loaded fiber samples as well. A more dense structure was formed in the case of NF-FUR-NaOH, while less dense supramolecular ordering was obtained in the case of NF-FUR-TEA. The latter refers to the furosemide-solubilizing effect of TEA, which increased the free volume holes between polymeric chains, enabling enhanced molecular mobility and so contributing to the plasticization of the system.

#### 3.2.4. Dissolution Study

**Figure 9:**
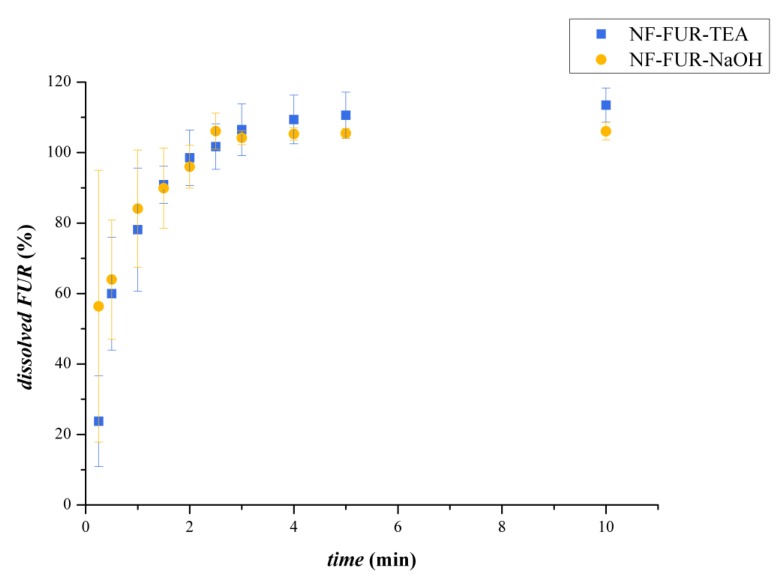
Comparison of in-vitro dissolution of TEA-(blue square label) or NaOH-containing (orange circle label) electrospun nanofibers.

The results of dissolution were summarized in Figure 9. The dissolution was rapid from both two different electrospun formulations and was complete in 3 min in each case.

## 4. Conclusions

Amorphous furosemide-salt-loaded electrospun nanofibers of similar morphology were formed, containing either sodium hydroxide or triethanolamine as solubility enhancers. The microstructure of the nanofibers revealed differences based on the *o*-Ps lifetime distributions, indicating various distributions of free volume holes. The latter indicate the different supramolecular arrangements of drug-loaded fibers forming highly or loosely packed structures, which could be of impact in the design of the composition of electrospun drug delivery systems with required physical-chemical characteristics.

## Figures and Tables

**Table 1:** Compositions (*w*/*w*%) of the two different aqueous polymeric solutions for the fiber formation.

Ingredients	Composition-TEA (*w/w*%)	Composition-NaOH (*w/w*%)
Polymer concentration *	15	17
Furosemide concentration	1	1
Triethanolamine concentration	1.5	-
NaOH (2 M concentration) solution concentration	-	~2
Purified water	82.5	~80

* Polymer composite ratio (HPC/PVP = 3/2).

**Table 2:** Summarized data on *o*-Ps lifetime distribution, as the *o*-Ps lifetime peak maximum, the max intensity (height) of *o*-Ps lifetime, full width at half maximum (FWHM) and the area under the curve (A).

Sample Name	*o*-Ps Lifetime (ns)	Height	FWHM	A
PM-FUR-TEA	1.29325	0.13188	0.08653	0.01215
PM-PLACEBO-TEA	1.3108	0.11264	0.09452	0.01133
NF-FUR-TEA	1.37286	0.08635	0.07866	0.00723
NF-PLACEBO-TEA	1.30485	0.06128	0.0869	0.00567
PM-FUR-NaOH	1.35086	0.15345	0.09621	0.01571
PM-PLACEBO-NaOH	1.31382	0.17043	0.09771	0.01773
NF-FUR-NaOH	1.33622	0.04768	0.10874	0.00552
NF-PLACEBO-NaOH	1.31933	0.06599	0.08334	0.00585
